# PLAG (1-Palmitoyl-2-Linoleoyl-3-Acetyl-rac-Glycerol) Modulates Eosinophil Chemotaxis by Regulating CCL26 Expression from Epithelial Cells

**DOI:** 10.1371/journal.pone.0151758

**Published:** 2016-03-24

**Authors:** Jinseon Jeong, Young-Jun Kim, Sun Young Yoon, Yong-Jae Kim, Joo Heon Kim, Ki-Young Sohn, Heung-Jae Kim, Yong-Hae Han, Saeho Chong, Jae Wha Kim

**Affiliations:** 1 Biomedical Translational Research Center, Korea Research Institute of Bioscience and Biotechnology, Daejeon 305–806, Republic of Korea; 2 Department of Functional Genomics, University of Science and Technology, Daejeon 305–806, Republic of Korea; 3 ENZYCHEM Lifesciences, KAIST-ICC, Daejeon 305–732, Republic of Korea; 4 Department of Pathology, Eulji University School of Medicine, Daejeon 302–120, Republic of Korea; University of Rochester Medical Center, UNITED STATES

## Abstract

Increased number of eosinophils in the circulation and sputum is associated with the severity of asthma. The respiratory epithelium produces chemokine (C-C motif) ligands (CCL) which recruits and activates eosinophils. A chemically synthesized monoacetyl-diglyceride, PLAG (1-palmitoyl-2-linoleoyl-3-acetyl-rac-glycerol) is a major constituent in the antlers of Sika deer (*Cervus nippon* Temminck) which has been used in oriental medicine. This study was aimed to investigate the molecular mechanism of PLAG effect on the alleviation of asthma phenotypes. A549, a human alveolar basal epithelial cell, and HaCaT, a human keratinocyte, were activated by the treatment of interleukin-4 (IL-4), and the expression of chemokines, known to be effective on the induction of eosinophil migration was analyzed by RT-PCR. The expression of IL-4 induced genes was modulated by the co-treatment of PLAG. Especially, CCL26 expression from the stimulated epithelial cells was significantly blocked by PLAG, which was confirmed by ELISA. The transcriptional activity of signal transducer and activator of transcription 6 (STAT6), activated by IL-4 mediated phosphorylation and nuclear translocation, was down-regulated by PLAG in a concentration-dependent manner. In ovalbumin-induced mouse model, the infiltration of immune cells into the respiratory tract was decreased by PLAG administration. Cytological analysis of the isolated bronchoalveolar lavage fluid (BALF) cells proved the infiltration of eosinophils was significantly reduced by PLAG. In addition, PLAG inhibited the migration of murine bone marrow-derived eosinophils, and human eosinophil cell line, EoL-1, which was induced by the addition of A549 culture medium.

## Introduction

Eosinophils are the main effector cells responsible for the severity of asthma. Infiltrated eosinophils in the bronchial mucosa cause damage to the airway epithelium and related nerves through the release of granule major basic proteins, lipid mediators, and reactive oxygen species [1]. Besides, eosinophils are a source of several molecules, such as TGF-α, TGF-ß, and FGF-2 [2–4], implicated in tissue remodeling processes. The consequences of excessive repair processes by eosinophils include deposition of extracellular matrix (ECM) proteins, smooth muscle increases, goblet-cell hyperplasia, and angiogenesis, which lead to airway hyper-responsiveness and airway obstruction [5]. Therefore, it is critical in the treatment of asthma to control eosinophil infiltration into the airway.

Asthma is a chronic inflammatory airway disease characterized by the infiltration of T cells, mast cells, and eosinophils into the respiratory region [6]. The clinical phenotype that defines allergic asthma is a coordinated product that results from the interactions between susceptible genes, external noxious materials, environment, defective barrier system, and immunological responses [7]. It is well known that CD4^+^ T-helper type lymphocytes play a prominent role in asthma, establishing T_H_2-skewed immune environments with coordinate production of T_H_2 cytokines, such as IL-4, IL-5 and IL-13, which drive inflammation associated with allergic responses through the recruitment and activation of T cells, eosinophils, and mast cells [8]. IL-4 is a pleiotrophic cytokine representing T_H_2 immunity. One of the important roles of IL-4 is to induce eosinophil attracting chemokines; chemokine (C-C motif) ligand 11, 24 and 26 (CCL11, 24 and 26; also known as Eotaxin-1, -2 and -3) [9–11].

The inflammation occurring in asthma is often described as eosinophilic [12–14]. During asthma progression, eosinophils are infiltrated into the airway in response to eosinophil chemotactic factors such as CCL11, 24 and 26, which are secreted by airway epithelial cells [15, 16]. CCL26 is the most potent eosinophil attracting factor and its increased level in the serum is correlated with the severity of asthma. While the expression of CCL11 and CCL24 is observed in the lung tissues of non-challenged asthmatic patients, CCL26 is only expressed in response to allergen challenge, indicating that CCL26 plays a distinct biological role from that of CCL11 and CCL24 [17]. In addition, the increased level of CCL26 is also associated with other kinds of eosinophilic diseases, such as atopic dermatitis, chronic rhinosinusitis, eosinophilic esophagitis and Churng-Strauss vasculitis [18]. Many pharmaceutical drugs for the treatment of asthma, for example glucocorticoids, are mainly targeting immunosuppression and anti-inflammatory effects. Dexamethasone (DEX), a synthetic glucocorticoid, diminished the induction of *CCL26* mRNA expression in human lung epithelial cells and dermal fibroblasts [17]. However, prolonged administration of this type of steroid medication triggers many side effects such as osteoporosis, hyperlipidemia, cardiovascular diseases, behavioral and cognitive changes, and gastritis and peptic ulceration [19]. The development of a therapeutic agent for the treatment of asthma without accompanying side effects is required.

1-Palmitoyl-2-linoleoyl-3-acetyl-rac-glycerol (PLAG) is a lipid molecule naturally occurring in a variety of seed oils, bovine udder, and deer horns. In traditional oriental medicine, extracts from the antlers of Sika deer (*Cervus nippon* Temminck) have been extensively used for alleviating various symptoms such as anorexia and fatigue, invigorating vital energy, and nourishing the blood [20]. In a murine model of ovalbumin (OVA)-induced asthma, PLAG reportedly alleviated the allergic asthma symptoms including the recruitment of inflammatory cells, methacholine responsiveness, mucus overproduction, and T_H_2 cytokine production [21]. It was also recently reported that PLAG modulates T_H_2 immunity by reducing the phosphorylation of signal transducer and activator of transcription 6 (STAT6), which inhibits the expression of affected genes such as IL-4 [22]. Although CCL26 is mainly produced in response to IL-4 via STAT6 pathway [23], little is known about the direct efficacy of PLAG on the regulation of CCL26. In this study, we discovered that PLAG can inhibit IL-4 induced CCL26 expression from epithelial cells by interfering with STAT6 signaling pathway, resulting in the control of eosinophil chemotaxis in an allergic asthma model. These findings suggest that PLAG can be developed as a therapeutic agent that ameliorates symptoms of asthma by suppressing CCL26 production from epithelial cells and the following eosinophil chemotaxis.

## Materials and Methods

### Reagents and cell culture

Human CCL26/CCL26 DuoSet ELISA kit was purchased from R&D systems (USA). Recombinant human IL-4, murine stem cell factor (SCF), murine FMS-like tyrosine kinase-3 (FLT3) ligand, and murine IL-5 were purchased from PeproTech (USA). Tri Reagent® was purchased from MRC Inc (USA). Attractene Transfection Reagent was purchased from QIAGEN Inc (USA). AS1517499 was purchased from Axon Medchem (USA). PLAG was obtained from Enzychem Lifesciences (Daejeon, South Korea), and diluted in dimethyl sulfoxide (DMSO, Sigma-Aldrich, USA) for the treatments. The same amount of DMSO were treated as negative control in every in vitro experiment. Anti-STAT6 and anti-phospho-STAT6 antibodies were purchased from Cell Signaling Technology (USA). Chicken OVA, aluminum potassium sulfate, and DEX were purchased from Sigma-Aldrich. PLAG was obtained from Enzychem Lifesciences Corporation (South Korea). A human keratinocyte cell line, HaCaT, and a human alveolar basal epithelial cell line, A549, were obtained from ATCC (USA) and cultured in DMEM and RPMI 1640 (Welgene, South Korea) respectively containing 10% FBS (Welgene), 100 U/mL penicillin, and 100 μg/mL streptomycin (Sigma-Aldrich) at 37°C in a humidified atmosphere with 5% CO_2_.

### RNA isolation and Reverse transcription polymerase chain reaction (RT-PCR)

Total RNAs from A549 and HaCaT were isolated using TRI Reagent® (MRC Inc., USA) according to the manufacturer’s instructions. 500 ng of total RNA was reverse-transcribed using primer (oligo-dT) and M-MLV RT enzyme (Promega, USA) for cDNA synthesis, followed by conventional PCR. The following primers were used in this study: 5’-AGTTGAAGGATGCGGGAGTA-3’ and 5’-TCTCCAGTTGAACATATCAAGCA-3’ for VCAM-1; 5’- GCCAGCGACTCCCCCACAAC-3’ and 5’- AAGCTGCGCCCGTTGTCCTC-3’ for ICAM-1; 5’- GTTCCTCTCCGAGCTCACC-3’ and 5’-GAGTTGTTCCAGCCCACATT-3’ and 5’- GAGTTGTTCCAGCCCACATT-3’ for MIF; 5’-GGCAGCCCTCGCTGTCATCC-3’ and 5’-GCCCTTCAAGGAGCGGGTGG-3’ for CCL5; 5’- AGCACCTGGACAAGAAAACCC-3’ and 5’- CCCCCATGAGGTAGAGAAGG-3’ for CCL7; 5’-AATGTCCCCAGAAAGCTGTG-3’ and 5’- TCATCTTTGCCAGGACCTTT-3’ for CCL11; 5’- GGAAAGCTCACACCCTGAAGA-3’ and 5’-CCAAACCAGCAACAAGTCAAATA-3’ for CCL13; 5’- AGGGACCTGCACACAGAGAC-3’ and 5’-AGGTAGTCCCGGGAGACAGT-3’ for CCL17; 5’- GCCTTCTGTTCCTTGGTGTC-3’ and 5’- TGTACCTCTGGACCCACTCC-3’ for CCL24; 5’- AATTGAGGCTGAGCCAAAGA-3’ and 5’- GGGTCCATGTAGCCTTCAGA-3’ for CCL26; 5’- CCATCACCATCTTCCAGGAG-3’ and 5’- ACAGTCTTCTGGGTGGCAGT-3’ for GAPDH.

### Enzyme-linked immunosorbent assay (ELISA)

For CCL26 immunoassay, A549 cells (0.5x10^6^ cells/mL) were seeded in 48-well plates (Corning, USA). The cells were pretreated with different doses of PLAG, AS1517499 and JAK I inhibitor followed by stimulation with IL-4 (10 ng/mL). After incubation, the supernatants were collected and analyzed by ELISA. The level of CCL26 was detected by Duo kit (R&D Systems, USA) according to the manufacturer’s instruction. Optical densities were measured at 450 nm using a Bio-Rad Model 550 microplate reader (Bio-Rad Laboratories, USA). The concentrations were calculated from a standard curve generated by a curve-fitting program.

The production of mouse CCL26 from mouse airway tissues was measured by ELISA using Eotaxin-3 ELISA kit (MyBiosource Inc., USA) according to the manufacturer’s instruction.

### Western blot analysis

Cells were lysed with RIPA buffer composed of 50 mM Tris-HCl (pH 7.4), 150 mM NaCl, 2 mM EDTA, 1% NP-40, 0.5% sodium deoxycholate, and 0.1% SDS containing protease inhibitor cocktail (Roche, USA) and Halt^TM^ phosphatase inhibitor (Thermo Scientific, USA). The lysate was heated in 5x protein sample buffer at 100°C for 15 min, separated by 10% SDS-PAGE, and transferred onto PVDF membranes (Millipore Corporation, USA). The membrane was blocked by 5% (w/v) bovine serum albumin (Bioworld, USA) in PBS containing 0.05% (v/v) Tween 20 (PBST) for 1 h and incubated overnight at 4°C with the primary antibody against STAT6 and phospho-STAT6 (1:1000; rabbit monoclonal IgG). Following the addition of horseradish peroxidase (HRP)-labeled secondary antibodies (Santa Cruz Biotechnology, USA) for 1 h, the blots were visualized using Immobilion Western Chemiluminescent HRP Substrate (Millipore Corporation).

### Transfection and luciferase assay

A549 cells (2x10^5^/mL) were seeded into 48-well plates and grown overnight. Transfection was performed by using Attractene transfection reagent according to the manufacturer’s instructions. Briefly, a total of 1–2 μg/well reporter luciferase plasmid containing four tandem copies of the STAT6 binding site (p4xSTAT6- Luc2P; Addgene, USA) was transfected. After 18 h, the cells were treated with different concentrations of PLAG for 1 h and then stimulated with IL-4 (10 ng/mL) for 24 h. Transient expression of the reporter gene was quantified using Dual-Glo® luciferase assay system (Promega) on the TD-20/20 Turner Luminometer (Promega).

### Confocal microscopy

A549 cells (2x10^5^ cells/mL) were seeded on a glass cover slip and grown overnight. The cells were treated with different concentrations of PLAG and stimulated with IL-4 (10 ng/mL) for 30 min. Cells were fixed for 20 min with 4% para-formaldehyde in PBS, permeabilized with methanol for 10 min, blocked with 1% BSA and incubated overnight with antibody against phospho-STAT6 at 4°C. Alexa Fluor 594-conjugated donkey antibody to rabbit (Life Technologies, USA) was used as a secondary antibody at 1:1000 dilution. Cover slips were washed, dried and mounted in Prolong Gold Antifade Reagent with DAPI (Roche) and visualized by Zeiss LSM 510 Meta (Zeiss, Germany) at ambient temperature. The images were processed and analyzed by Image J (NIH, USA).

### Animals

Specific pathogen-free Female BALB/c mice (6–8 weeks of age) were purchased from Koatech Corporation (South Korea). Mice were housed in a specific pathogen-free facility under consistent temperature and light cycles. All experimental procedures were approved by the Institutional Animal Care and Use Committee of the Korea Research Institute of Bioscience and Biotechnology performed in compliance with the National Institutes of Health Guidelines for the care and use of laboratory animals and Korean national laws for animal welfare.

### Mouse allergic asthma

Mice were divided into six groups (n = 3 for each group). To induce allergic asthma, the mice were sensitized by injecting 20 μg of chicken egg albumin (OVA, Sigma-Aldrich) mixed with 2 mg of aluminum potassium sulfate (Sigma-Aldrich) in 100 μL of PBS intraperitoneally (i.p.) on day 0 and 14. From day 15 to 21, different doses of PLAG (10, 50, 250 mg/Kg) or DEX (3 mg/Kg) were administrated to the mice by oral gavage daily after being emulsified by flushing thoroughly through three-way valves. On day 20, the mice were anaesthetized with i.p. injection of 2,2,2-Tribromoethanol (Avertin, Sigma-Aldrich) dissolved in tert-Amyl alcohol (500 mg/Kg) and then challenged with 100 μg of OVA in 50μl PBS intranasally (i.n.). Forty-eight-hour after OVA challenge, the mice were sacrificed by an i.p. injection of pentobarbital (Hanlim Pharm. C., South Korea), and tracheostomy was performed. To obtain bronchoalveolar lavage fluid (BALF), 0.75mL of ice-cold PBS was infused to the lung and withdrawn via tracheal cannulation two times. Immune cell populations in BALFs were analyzed by complete blood count (CBC) using a hematology analyzer, Mindray BC-5300 (China).

### Flow cytometry

BALF cells or BM-derived eosinophils were resuspended with FACS buffer (1% FBS in PBS) and stained with Siglec-F-PE and CD11b PE-Cy7 (BD Biosciences, USA) for 30min at 4°C. Then the cells were washed with FACS buffer twice and analyzed by FACS CANTO flow cytometer (BD Biosciences). FACS data were processed by Flow Jo software (Tree Star, USA).

### Histopathology

Immediately after collecting BALF samples, lung tissues were incised and fixed in ice-cold 10% formalin. Each tissue was embedded in paraffin, sectioned at 4-μm thickness, and deparaffinized after being mounted on a slide glass. The slides were stained with hematoxylin and eosin (H&E) solution, and examined under light microscopy (Olympus, Japan).

### Murine eosinophils and migration assay

Murine eosinophils were differentiated from bone marrow (BM) cells isolated from BALB/c mice. Briefly, BM cells were collected from the femurs of BALB/c mice and cultured in RPMI 1640 containing 20% FBS and 100 U/mL penicillin/streptomycin. 80 ng/mL of SCF and FLT3 ligand were treated to the medium during initial culture. On day 4, cells were transferred to a medium containing 10 ng/mL of IL-5 and subcultured every other day up to day 14, maintaining cell density at 1x10^6^ cells/mL.

A549 cells were pretreated with different concentrations of PLAG for 1h and stimulated with IL-4 for 72 h. The supernatants were used for migration assay. The differentiated eosinophils (2x10^5^ cells/100μL) and EoL-1 cells (1x10^6^ cells/100μL) were added to the upper chamber of Transwell 24-well plates with 5 μm polycarbonate membrane filters (Falcon, USA). Six hundred microliters of CCL26-containing supernatant was added to the bottom chamber and incubated for overnight. The total numbers of migrated eosinophils were counted by trypan blue staining.

### Cell proliferation assay

Cell viability was checked by colorimetric water-soluble tetrazolium salt (WST) assay using EZ-CYTOX (DaeilLab Service, South Korea) according to the manufacturer’s instructions. Briefly, A549 cells (5x10^4^ cells/well) were seeded in 96-well plates, pretreated with different doses of PLAG, and then stimulated with IL-4 (10 ng/mL) for 72 h. The number of viable cells was measured at 450 nm of the absorbance using a Bio-Rad Model 550 microplate reader.

### Statistical Analysis

The significance of differences between experimental groups was analyzed using Student’s unpaired t-test. Mean values from three different samples were depicted in the graph. Differences in means were considered significant if *p*<0.05. Each bar represents the mean ± SD.

### Analysis of *CCL26* expression in mouse tissues

Lung inflammation was induced by intranasal injection of LPS (25 mg/Kg) into Balb/c mice (7 week, male) for 15 h. PBS was injected intranasally as negative control. Mice were sacrificed by cervical dislocation and lung tissues were isolated. Total RNAs were purified from the lung tissues followed by RT-PCR analysis as described in ‘Materials and Method’. Primer sequences were mCCL26-F; 5’-CTTCGATTTGGGTCTCCTTG-3’, and mCCL26-R; 5’-TCACTGGTGCAGCTCTTGTC-3’. The PCR product was purified and analyzed by sequencing (Macrogen, South Korea).

## Results

### *PLAG* inhibits *IL-4* induced *CCL26* expression

**Fig 1:**
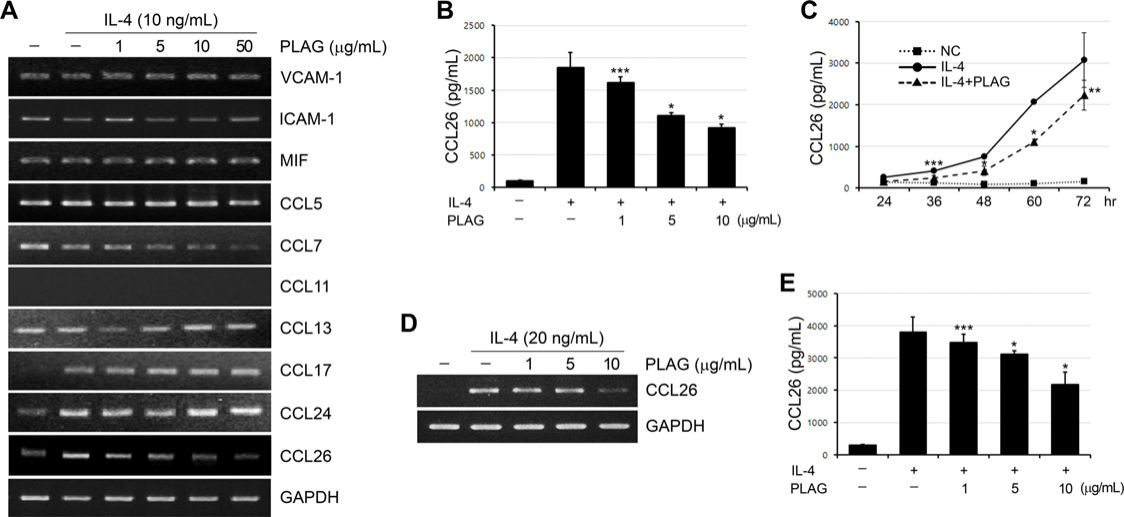
IL-4 induced CCL26 expression is inhibited by PLAG. (A) A549 cells were treated with various doses of PLAG (1, 5, 10, 50 μg/mL) for 1 h and stimulated with IL-4 (10 ng/mL) for 24 h. The mRNA levels of following genes, VCAM-1, ICAM-1, MIF, CCL5, CCL7, CCL11, CCL13, CCL17, CCL24, and CCL26 were analyzed by conventional RT-PCR. Each reaction was repeated three times and representative images were displayed. GAPDH was used as an internal control. (B) The culture supernatant was harvested at 48 h, and the protein level of CCL26 was evaluated by ELISA. PLAG decreased CCL26 secretion from A549 cells in a dose-dependent manner. *p<0.001, ***p<0.05. (C) A549 cells were pretreated with PLAG (10 μg/mL) for 1 h and stimulated with IL-4 (10 ng/mL) for various lengths of time. PLAG decreased CCL26 secretion from A549 cells from the early time point. NC; negative control. *p<0.001, **p<0.01, *** *p*<0.05. (D) HaCaT cells were treated with various doses of PLAG and stimulated with IL-4 (20 ng/mL) for 24 h. The mRNA levels of CCL 26 were analyzed by RT-PCR. (E) PLAG also decreased CCL26 secretion from HaCaT cells in a dose-dependent manner. *p<0.001, ***p<0.05.

To find the relationship of PLAG function in asthmatic pathogenesis, we analyzed the expression of various genes known to be involved in the regulation of eosinophil migration by conventional RT-PCR. The expression of CCL26 was distinguished from other genes in that it was significantly inhibited by PLAG in a dose-dependent manner (Fig 1A). Two of the other Eotaxin family member, CCL11 and CCL24, and adhesion molecules, VCAM-1 and ICAM-1, were not expressed in response to IL-4 stimulation nor affected by PLAG. Macrophage migration inhibitory factor (MIF), CCL5 (RANTES) and CCL13 (MCP-4), which are known to induce eosinophil infiltration into lesional sites [24–26], also showed no significant changes by IL-4 and PLAG. The expression of CCL17 was induced by IL-4, but was not affected by PLAG. Interestingly, the expression of CCL7 was synergistically diminished by IL-4 and PLAG. The effect of PLAG on IL-4 induced CCL26 secretion in A549 cells was also verified by ELISA. PLAG caused a concentration- and time-dependent inhibition of IL-4 induced CCL26 production from the epithelial cells (Fig 1B and 1C). PLAG effect on CCL26 expression was confirmed in the other epithelial cell line, HaCaT (Fig 1D and 1E). These findings suggest that PLAG is able to inhibit IL-4 induced production of CCL26 from epithelial cells.

### *PLAG* inhibits transcriptional activity of *STAT6*

**Fig 2:**
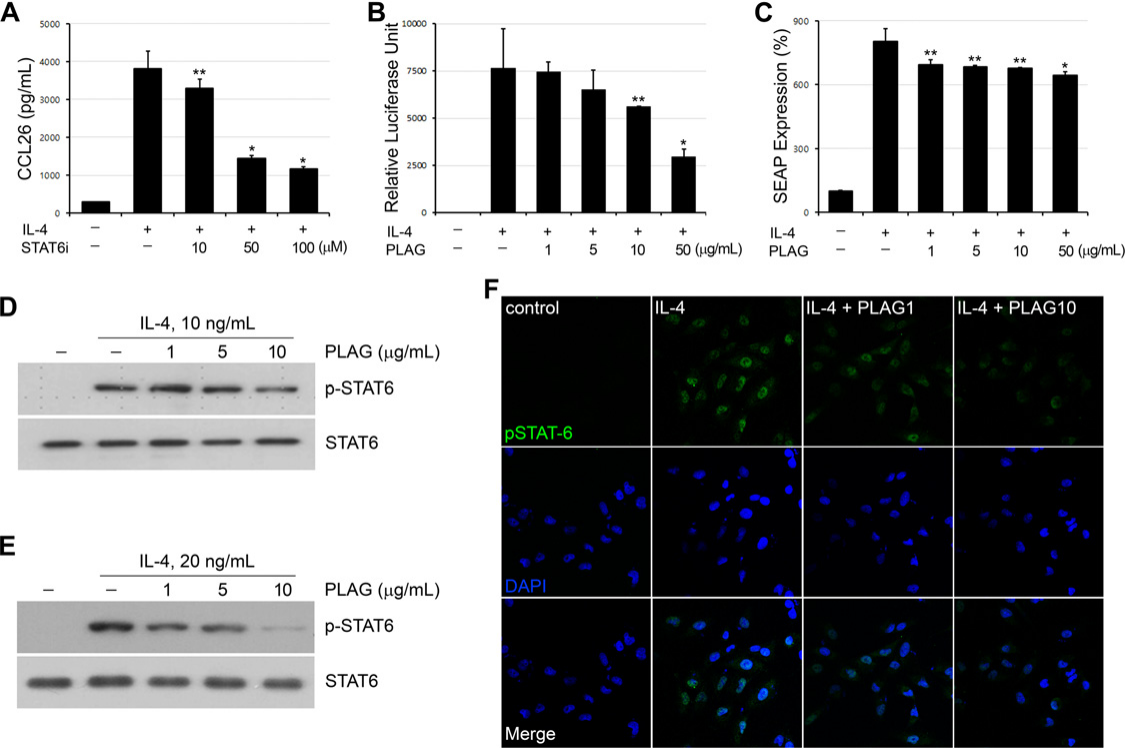
PLAG inhibits IL-4 induced STAT6 phosphorylation. (A) Various concentration of a STAT6 inhibitor (STAT6i, AS1517499) were treated onto A549 cells which were activated by IL-4 (10 ng/mL), and showed inhibitory effect on the secretion of CCL26 from the epithelial cells in a concentration-dependent manner. *p<0.001, **p<0.01. (B) Reporter construct containing luciferase gene regulated by STAT6 activity was transfected into A549 cells and the effect of PLAG on the expression of the luciferase gene was analyzed by Dual-Glo luciferase system. The inhibitory effect of PLAG on STAT6 activity resulted in the decrease of luciferase expression. *p<0.001, **p<0.01. (C) The effect of PLAG on the inhibition of IL-4-induced SEAP activity was evaluated in HEK Blue IL-4/IL-13 cells. *p<0.001, **p<0.01. (D-E) Cellular extracts of IL-4 and/or PLAG treated epithelial cells were analyzed by Western blotting. PLAG decreased the phosphorylation of STAT6 with dose dependency in both A549 (D) and HaCaT (E). (F) Confocal microscopy showed that IL-4 induced nuclear localization of phosphorylated STAT6, which was inhibited by the co-treatment of PLAG.

It is well known that IL-4 induced CCL26 expression is mediated by JAK1/STAT6 signaling pathway. We confirmed the signaling pathway of CCL26 expression in the epithelial cells by treating with a STAT6 inhibitor, AS1517499. IL-4 induced production of CCL26 was decreased by AS1517499 (Fig 2A). An unspecified JAK inhibitor, JAK inhibitor I, also exhibited suppressive effect on IL-4 induced CCL26 expression (data not shown). Promoter analysis using a reporter construct containing STAT6-binding domains confirmed the modulatory effect of PLAG on the transcriptional activity of STAT6 in the epithelial cells (Fig 2B). Transcriptional activity of STAT6 was remarkably inhibited by PLAG in a dose-dependent manner. Additional reporter analysis using HEK Blue IL-4/IL-13 cell supported PLAG effect on the regulation of STAT6 activities (Fig 2C). The phosphorylation of STAT6 was effectively inhibited by PLAG dose-dependently (Fig 2D and 2E). In addition, PLAG decreased the nuclear translocation of phosphorylated STAT6 (Fig 2F). These data indicate that PLAG is able to block STAT6 phosphorylation and transcriptional activities resulting in the decrease of CCL26 expression.

### Administration of *PLAG* attenuates asthma phenotypes in an allergic asthma model

**Fig 3:**
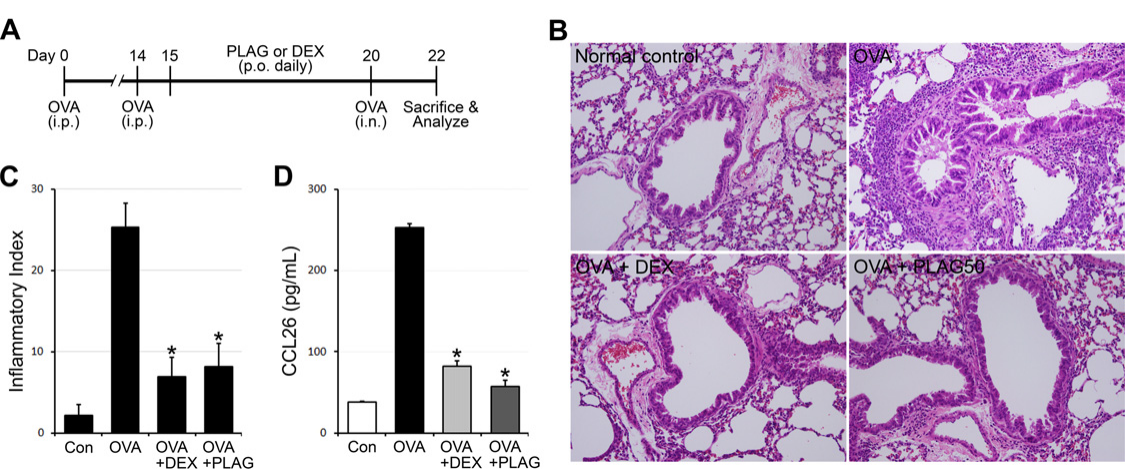
PLAG alleviates inflammatory phenotypes in allergic asthma model. (A) Mice were sensitized and challenged by OVA, and the efficacy of PLAG and DEX was tested in the generated asthma model as depicted. (B-C) The lung tissues from the OVA-sensitized and–challenged mice were stained with H&E, and the representative images were displayed (X200 magnification, B). Infiltrated immune cells were counted, calculated and displayed as inflammation index. Eosinophils from each of the three mice per treatment group were analyzed morphometrically and mean eosinophil counts per 50 × 50 μm area ± SD were displayed. Four such areas were measured from each tissue section. PLAG was the most effective with a dose of 50 mg/Kg as showed in (B) and calculated in (C). *p<0.001. (D) The expression level of CCL26 in BALFs of OVA-challenged mice was analyzed by ELISA. Each sample was analyzed in triplicate and displayed as mean ± SD. *p<0.001.

To test the effect of PLAG-mediated CCL26 inhibition on the regulation of eosinophil infiltration into airway inflammatory sites, PLAG was administered per orally into OVA-sensitized and -challenged mice (Fig 3A). DEX was treated as a comparative drug. A large number of blood cells including eosinophils were infiltrated into the lung tissues of OVA-challenged mice compared with the control group, which was administered with PBS (Fig 3B and 3C). Most of infiltrated cells were concentrated at the peribronchiolar and perivascular lesions. The infiltration of immune cells was decreased by PLAG administration similarly with DEX treatment. CCL26 concentration in bronchoalveolar lavage fluid (BALF) was increased in OVA-challenged mice, which was decreased by DEX or PLAG administration (Fig 3D).

**Fig 4:**
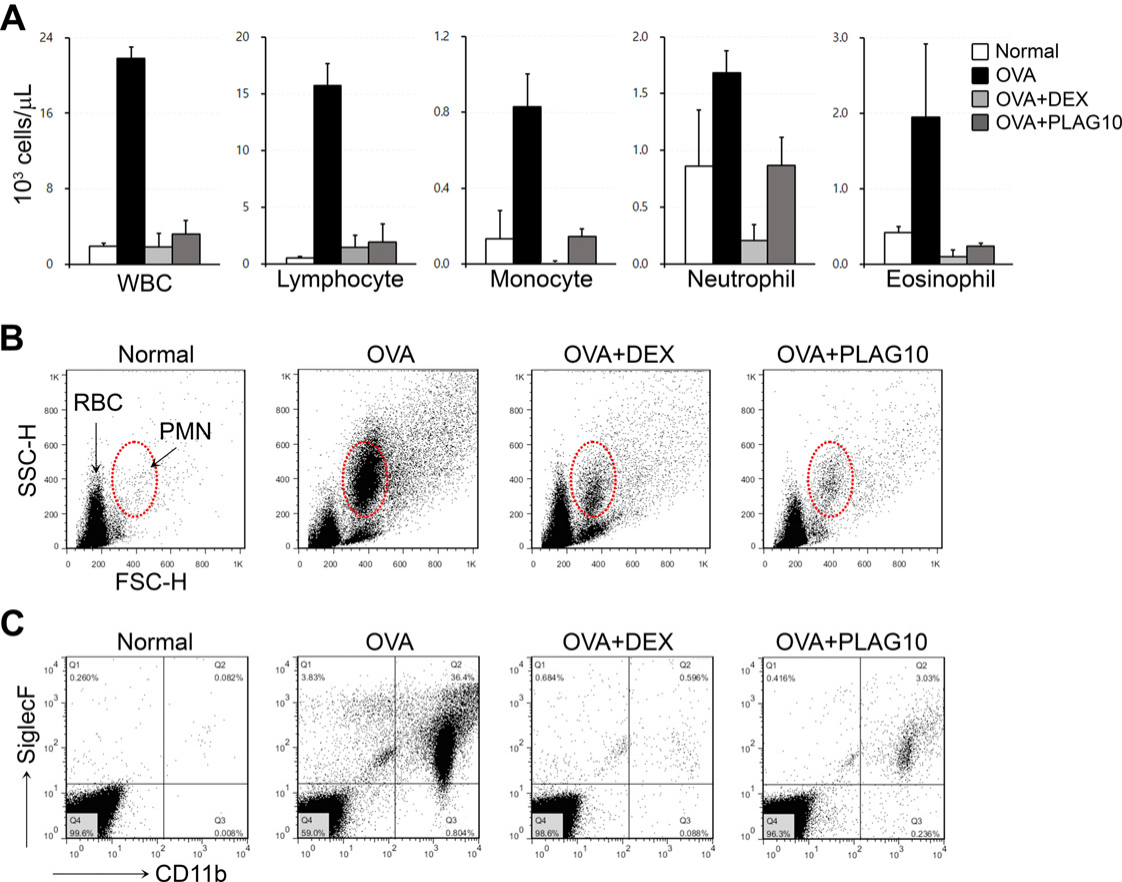
PLAG decreases eosinophil infiltration into the airways of asthmatic mice. (A) Cells were harvested from the BALFs of OVA-challenged mice and analyzed by CBC. The number of infiltrated immune cells were increased in OVA-challenged mice. PLAG or DEX treatment inhibited immune cell infiltration into airways to the level of normal control. (B) After CBC, cells were collected from the BALF, and analyzed by flow cytometry. PLAG and DEX treatment induced the recovery of PMN cell populations which were increased in the allergic mice. (C) SiglecF+/CD11b+ eosinophils were increased in the OVA-challenged mice, which were decreased in PLAG- or DEX-treated mice.

CBC analysis of the BALFs showed that most blood cell populations were massively increased in OVA-challenged mice, which were decreased by PLAG or DEX treatment (Fig 4A). Especially, the number of infiltrated eosinophils into the airway of OVA-challenged mice was decreased in PLAG-treated mice (Fig 4A). FACS analysis also confirmed the inhibitory effect of PLAG on the infiltration of eosinophils into inflammatory airways. PLAG or DEX administration decreased the number of polymorphonuclear (PMN) cells (Fig 4B) or SiglecF+/CD11b+ eosinophils (Fig 4C) in the BALFs from OVA-challenged mice.

### *PLAG* inhibits eosinophil migration by suppressing *CCL26* expression

**Fig 5:**
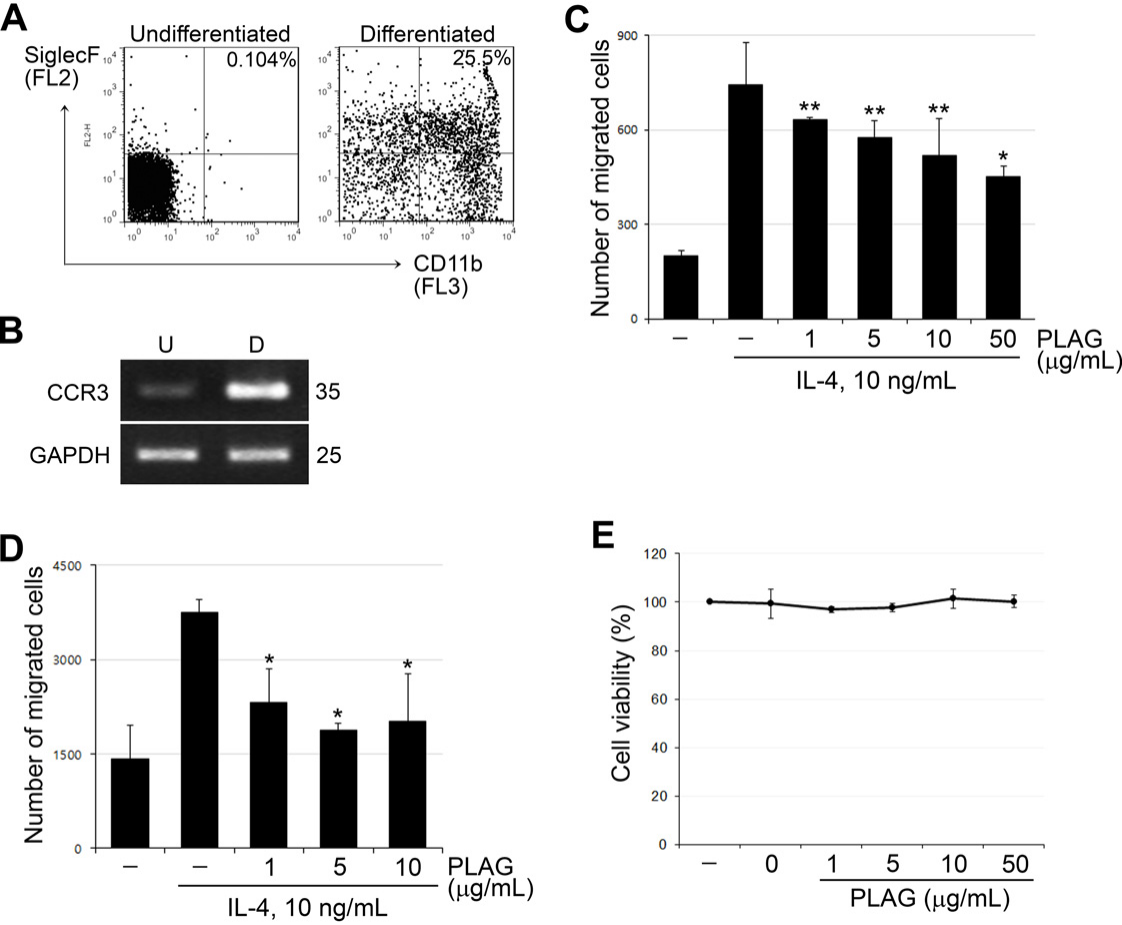
PLAG inhibits eosinophil migration in vitro. (A) Murine eosinophils were differentiated from BM cells by treating SCF, FLT3 ligand and IL-5 for 2w. The differentiated cells were analyzed by FACS staining with SiglecF and CD11b. SiglecF+/CD11b+ cells were increased in the differentiated cells to ~25%. (B) CCR3 expression was increased in the differentiated murine eosinophils. (C-D) The supernatant of IL-4 and/or PLAG-treated A549 cells was prepared and used for the migration assay as described in Materials and Methods. The transmigration of eosinophil was decreased by PLAG treatment in both murine BM-derived eosinophils (C) and human eosinophil cell line, EoL-1 (D). *p<0.001, **p<0.01. (E) PLAG did not exhibit any cytotoxic effects on A549 cells at various concentrations.

To explore whether PLAG is directly able to inhibit eosinophil migration, we performed a chemotaxis assay using murine eosinophils differentiated from bone marrow cells and a human eosinophil cell line, EoL-1. Differentiation of eosinophils from mouse bone marrow cells was verified by analyzing surface presentation of eosinophil specific markers in the differentiated cells. SiglecF+/CD11b+ cells were increased in the differentiated murine eosinophils (Fig 5A). In addition, the expression of C-C chemokine receptor 3 (CCR3), a cell surface receptor for CCL26, was also increased in differentiated murine eosinophils (Fig 5B). CCL26-containing medium from IL-4 stimulated A549 cells caused chemotactic invasion of differentiated murine eosinophils (Fig 5C), which was inhibited by pre-treatment of PLAG in a concentration-dependent manner. The invasion of EoL-1, a human eosinophil, was also inhibited by PLAG in a similar pattern (Fig 5D). Any cytotoxic effects were not detected in A549 cells by PLAG treatment (Fig 5E). These findings suggest that the decrease of CCL26 production from epithelial cells by PLAG led to the suppression of eosinophil migration.

## Discussion

In the Orient, many natural products have been used as medical herbs for the treatment of asthma, but the modes of action of such medicines are often unclear [27]. In this study, we discovered that an active component of deer’s antlers can exerts its pharmacological effects on the inhibition of eosinophil chemotaxis. PLAG inhibits the phosphorylation and activation of STAT6 transcription factor, which results in the down-regulation of an eosinophil chemotactic molecule, CCL26.

Eosinophil infiltration into the allergic inflammatory tissues is mediated by a combination of various processes. Chemokine ligands for CCR3 are important eosinophil chemotactic factors because this receptor is strongly expressed by eosinophils. Reported ligands for this receptor include RANTES (CCL5), MCP-3 (CCL7), MCP-2 (CCL8), eotaxin-1 (CCL11), MCP-4 (CCL13), TARC (CCL17), eotaxin-2 (CCL24), and eotaxin-3 (CCL26). Our RT-PCR data showed that CCL26 is effectively inhibited by PLAG treatment (Fig 1A). The expression of CCL7 was also inhibited by PLAG although the inductive effect of IL-4 was not detected. CCL7 has been identified as a monocyte attractant produced from certain tumor cells and macrophage [28], and shown to function as a chemotactic for eosinophil infiltration into the lung tissues in T_H_2-type pulmonary granuloma, which was blocked by anti-IL-4 treatment [29]. In A549 lung epithelial cells, IL-4 and PLAG treatment synergistically blocked CCL7 expression (Fig 1A). The experimental analysis of PLAG effect on CCL7 expression and eosinophil transmigration in asthma model would be informative for identifying the functional mechanism of PLAG completely.

The expression of Eotaxin family is regulated by two separate pathways, which induce the activation of STAT6 or NF-κB respectively [30, 31]. IL-4/IL-13 simulated pathway causes the activation of STAT6 to induce eotaxin, mainly CCL26 (Eotaxin-3) expression, while TNF-α/IFN-γ mediated pathway activate NF-κB of which the main target is CCL11 (Eotaxin-1). According to an OVA-induced asthma model experiment using STAT6-/- mice, airway eosinophilia was blocked by reducing eotaxin level in the pulmonary tissues [32]. The pulmonary eosinophilia in STAT6-/- mice was reconstituted by the intranasal administration of recombinant eotaxin. In addition, STAT6-/- eosinophils have reduced migratory activities and are unable to induce the development of allergic airway inflammation [33]. These data suggest STAT6 is a master regulator of eosinophil transepithelial migration by regulating both the production of Eotaxin from epithelial cells and the responsiveness of eosinophils to Eotaxin. STAT6 regulatory activity of PLAG can be used to modulate eosinophil chemotaxis in inflammatory environments. We have demonstrated that PLAG inhibits the invasion of eosinophils in vitro (Fig 5), and the infiltration of eosinophils in in vivo model (Figs 3 and 4). The study of intracellular signaling mechanism has demonstrated PLAG regulates the transcriptional activity of STAT6 to modulated CCL26 expression (Fig 2). Additionally, PLAG has been reported to modulate T_H_2 immunity by regulating the expression of IL-4 which is also controlled by STAT6 [22]. We could conclude that PLAG is effective in immune-modulation by regulating STAT6 activity, which result in the control of T_H_2 immunity and eosinophil transmigration.

The distinctive up-regulation of CCL26 makes this chemokine an important mediator for eosinophil recruitment in chronic allergic asthma. In mice, CCL26 has been identified as a pseudogene, which exhibits a significant sequence homology with human CCL26 [34]. To verify the expression of CCL26 in mice, we have thoroughly analyzed RNA transcript by RT-PCR using several primer pairs, and discovered murine CCL26 expression was induced by inflammation (S1 Fig). Recent study using a mouse model has showed that intraperitoneal injection of recombinant human CCL26 induced rapid recruitment of mouse eosinophils [35]. In addition, the increase of CCL26 protein in the BALF of OVA-induced asthmatic mice has been reported recently [27]. Our eosinophil migration assay data verified that human CCL26 is active on the induction of mouse eosinophil migration demonstrating there are cross reactivity between human CCL26 and its mouse counterpart, CCR3, which is a receptor for Eotaxin family proteins in granulocytes (Fig 5).

Many studies have shown that the production of CCL26 involves JAK1/STAT6 signaling pathway [23, 36, 37]. We observed that PLAG diminishes STAT6 phosphorylation in the cytoplasm and its transcriptional activity in the nucleus. It is less possible that PLAG bind directly with STAT6 molecules to interfere its phosphorylation because PLAG is a lipid molecule. Instead, it may regulate upstream signaling molecules such as JAK1 and protein kinase C (PKC) [38]. In conclusion, PLAG inhibited the production of CCL26 from the epithelial cells by regulating IL-4/JAK1/STAT6 signaling pathway modulating the transmigration of eosinophils. Therefore, we expect that PLAG could be developed as a therapeutic agent for asthma treatment.

## Supporting Information

S1 Fig The expression of CCL26 was detected in the lung tissues from LPS-challenged mice. **A**. Coding sequence for mouse CCL26 was depicted, and primer sequences were marked with bottom line. Mouse CCL26 has been predicted to contain three exons, which was displayed in color codes; blue for exon1, orange for exon2, and green for exon3. **B**. The expression of mouse CCL26 was induced by LPS challenge. **C**. DNA sequence of PCR product in B was compared with the predicted sequence (NM_001013412), which showed 100% homology.
(PSD)

## Abbreviations

PLAG: 1-palmitic acid-2-linoleic acid-3-acetyl-rac-glycerol
CCL26: chemokine (C-C motif) ligand 26
CCR3: C-C chemokine receptor 3
IL-4: interleukin 4
STAT6: signal transducer and activator of transcription 6

## References

[1] Kay AB, Phipps S, Robinson DS. A role for eosinophils in airway remodelling in asthma. Trends Immunol. 2004;25(9):477–82. 10.1016/j.it.2004.07.006 . 15324740

[2] Egesten A, Calafat J, Knol EF, Janssen H, Walz TM. Subcellular localization of transforming growth factor-alpha in human eosinophil granulocytes. Blood. 1996;87(9):3910–8. . 8611720

[3] Duvernelle C, Freund V, Frossard N. Transforming growth factor-beta and its role in asthma. Pulm Pharmacol Ther. 2003;16(4):181–96. 10.1016/S1094-5539(03)00051-8 . 12850120

[4] Hoshino M, Takahashi M, Aoike N. Expression of vascular endothelial growth factor, basic fibroblast growth factor, and angiogenin immunoreactivity in asthmatic airways and its relationship to angiogenesis. J Allergy Clin Immunol. 2001;107(2):295–301. 10.1067/mai.2001.111928 . 11174196

[5] Humbles AA, Lloyd CM, McMillan SJ, Friend DS, Xanthou G, McKenna EE, et al A critical role for eosinophils in allergic airways remodeling. Science. 2004;305(5691):1776–9. 10.1126/science.1100283 . 15375268

[6] Lambrecht BN, Hammad H. The immunology of asthma. Nat Immunol. 2015;16(1):45–56. 10.1038/ni.3049 . 25521684

[7] Bel EH. Clinical phenotypes of asthma. Curr Opin Pulm Med. 2004;10(1):44–50. . 14749605 10.1097/00063198-200401000-00008

[8] Robinson DS, Hamid Q, Ying S, Tsicopoulos A, Barkans J, Bentley AM, et al Predominant TH2-like bronchoalveolar T-lymphocyte population in atopic asthma. N Engl J Med. 1992;326(5):298–304. 10.1056/NEJM199201303260504 . 1530827

[9] Zhou X, Hu H, Balzar S, Trudeau JB, Wenzel SE. MAPK regulation of IL-4/IL-13 receptors contributes to the synergistic increase in CCL11/eotaxin-1 in response to TGF-beta1 and IL-13 in human airway fibroblasts. J Immunol. 2012;188(12):6046–54. 10.4049/jimmunol.1102760 22573806 PMC3370069

[10] Lezcano-Meza D, Davila-Davila B, Vega-Miranda A, Negrete-Garcia MC, Teran LM. Interleukin (IL)-4 and to a lesser extent either IL-13 or interferon-gamma regulate the production of eotaxin-2/CCL24 in nasal polyps. Allergy. 2003;58(10):1011–7. . 14510718 10.1034/j.1398-9995.2003.00174.x

[11] Kagami S, Saeki H, Komine M, Kakinuma T, Tsunemi Y, Nakamura K, et al Interleukin-4 and interleukin-13 enhance CCL26 production in a human keratinocyte cell line, HaCaT cells. Clin Exp Immunol. 2005;141(3):459–66. 10.1111/j.1365-2249.2005.02875.x 16045735 PMC1809447

[12] Lovett CJ, Whitehead BF, Gibson PG. Eosinophilic airway inflammation and the prognosis of childhood asthma. Clin Exp Allergy. 2007;37(11):1594–601. 10.1111/j.1365-2222.2007.02839.x . 17949371

[13] Bousquet J, Chanez P, Lacoste JY, Barneon G, Ghavanian N, Enander I, et al Eosinophilic inflammation in asthma. N Engl J Med. 1990;323(15):1033–9. 10.1056/NEJM199010113231505 . 2215562

[14] Barnes PJ. Immunology of asthma and chronic obstructive pulmonary disease. Nat Rev Immunol. 2008;8(3):183–92. 10.1038/nri2254 . 18274560

[15] Chae SC, Lee YC, Park YR, Shin JS, Song JH, Oh GJ, et al Analysis of the polymorphisms in eotaxin gene family and their association with asthma, IgE, and eosinophil. Biochem Biophys Res Commun. 2004;320(1):131–7. 10.1016/j.bbrc.2004.05.136 . 15207712

[16] Dulkys Y, Schramm G, Kimmig D, Knoss S, Weyergraf A, Kapp A, et al Detection of mRNA for eotaxin-2 and eotaxin-3 in human dermal fibroblasts and their distinct activation profile on human eosinophils. J Invest Dermatol. 2001;116(4):498–505. 10.1046/j.1523-1747.2001.01299.x . 11286614

[17] Banwell ME, Tolley NS, Williams TJ, Mitchell TJ. Regulation of human eotaxin-3/CCL26 expression: modulation by cytokines and glucocorticoids. Cytokine. 2002;17(6):317–23. 10.1006/cyto.2002.1021 . 12061839

[18] Larose MC, Chakir J, Archambault AS, Joubert P, Provost V, Laviolette M, et al Correlation between CCL26 production by human bronchial epithelial cells and airway eosinophils: Involvement in patients with severe eosinophilic asthma. J Allergy Clin Immunol. 2015 10.1016/j.jaci.2015.02.039 . 25936567

[19] Moghadam-Kia S, Werth VP. Prevention and treatment of systemic glucocorticoid side effects. Int J Dermatol. 2010;49(3):239–48. 10.1111/j.1365-4632.2009.04322.x 20465658 PMC2872100

[20] Yang HO, Park JS, Cho SH, Yoon JY, Kim MG, Jhon GJ, et al Stimulatory effects of monoacetyldiglycerides on hematopoiesis. Biol Pharm Bull. 2004;27(7):1121–5. . 15256752 10.1248/bpb.27.1121

[21] Shin IS, Shin NR, Jeon CM, Kwon OK, Sohn KY, Lee TS, et al EC-18, a synthetic monoacetyldiglyceride (1-palmitoyl-2-linoleoyl-3-acetylglycerol), attenuates the asthmatic response in an aluminum hydroxide/ovalbumin-induced model of asthma. Int Immunopharmacol. 2014;18(1):116–23. 10.1016/j.intimp.2013.11.006 . 24269625

[22] Yoon SY, Kang HB, Ko YE, Shin SH, Kim YJ, Sohn KY, et al 1-palmitoyl-2-linoleoyl-3-acetyl-rac-glycerol (EC-18) Modulates Th2 Immunity through Attenuation of IL-4 Expression. Immune Netw. 2015;15(2):100–9. 10.4110/in.2015.15.2.100 25922599 PMC4411508

[23] Hoeck J, Woisetschlager M. Activation of eotaxin-3/CCLl26 gene expression in human dermal fibroblasts is mediated by STAT6. J Immunol. 2001;167(6):3216–22. . 11544308 10.4049/jimmunol.167.6.3216

[24] Yoshihisa Y, Makino T, Matsunaga K, Honda A, Norisugi O, Abe R, et al Macrophage migration inhibitory factor is essential for eosinophil recruitment in allergen-induced skin inflammation. J Invest Dermatol. 2011;131(4):925–31. 10.1038/jid.2010.418 . 21191413

[25] Venge J, Lampinen M, Hakansson L, Rak S, Venge P. Identification of IL-5 and RANTES as the major eosinophil chemoattractants in the asthmatic lung. J Allergy Clin Immunol. 1996;97(5):1110–5. . 8626989 10.1016/s0091-6749(96)70265-8

[26] Garcia-Zepeda EA, Combadiere C, Rothenberg ME, Sarafi MN, Lavigne F, Hamid Q, et al Human monocyte chemoattractant protein (MCP)-4 is a novel CC chemokine with activities on monocytes, eosinophils, and basophils induced in allergic and nonallergic inflammation that signals through the CC chemokine receptors (CCR)-2 and -3. J Immunol. 1996;157(12):5613–26. . 8955214

[27] Wang J, Zhang T, Ma C, Wang S. Puerarin attenuates airway inflammation by regulation of eotaxin-3. Immunol Lett. 2015;163(2):173–8. 10.1016/j.imlet.2014.12.002 . 25530546

[28] Opdenakker G, Froyen G, Fiten P, Proost P, VD J. Human monocyte chemotactic protein-3 (MCP-3): molecular cloning of the cDNA and comparison with other chemokines. Biochem Biophys Res Commun. 1993;191(2):535–42. 8461011 10.1006/bbrc.1993.1251

[29] Shang XZ, Chiu BC, Stolberg V, Lukacs NW, Kunkel SL, Murphy HS, et al Eosinophil recruitment in type-2 hypersensitivity pulmonary granulomas: source and contribution of monocyte chemotactic protein-3 (CCL7). Am J Pathol. 2002;161(1):257–66. 12107110 10.1016/S0002-9440(10)64177-6 PMC1850678

[30] Matsukura S, Stellato C, Plitt JR, Bickel C, Miura K, Georas SN, et al Activation of eotaxin gene transcription by NF-kappa B and STAT6 in human airway epithelial cells. J Immunol. 1999;163(12):6876–83. 10586089

[31] Yuan Q, Campanella GS, Colvin RA, Hamilos DL, Jones KJ, Mathew A, et al Membrane-bound eotaxin-3 mediates eosinophil transepithelial migration in IL-4-stimulated epithelial cells. Eur J Immunol. 2006;36(10):2700–14. 16983721 10.1002/eji.200636112

[32] Hoshino A, Tsuji T, Matsuzaki J, Jinushi T, Ashino S, Teramura T, et al STAT6-mediated signaling in Th2-dependent allergic asthma: critical role for the development of eosinophilia, airway hyper-responsiveness and mucus hypersecretion, distinct from its role in Th2 differentiation. Int Immunol. 2004;16(10):1497–505. 15351784 10.1093/intimm/dxh151

[33] Stokes K, LaMarche NM, Islam N, Wood A, Huang W, August A. Cutting edge: STAT6 signaling in eosinophils is necessary for development of allergic airway inflammation. J Immunol. 2015;194(6):2477–81. 10.4049/jimmunol.1402096 25681342 PMC4470169

[34] Pope SM, Fulkerson PC, Blanchard C, Akei HS, Nikolaidis NM, Zimmermann N, et al Identification of a cooperative mechanism involving interleukin-13 and eotaxin-2 in experimental allergic lung inflammation. J Biol Chem. 2005;280(14):13952–61. 10.1074/jbc.M406037200 . 15647285

[35] Nakayama T, Watanabe Y, Oiso N, Higuchi T, Shigeta A, Mizuguchi N, et al Eotaxin-3/CC chemokine ligand 26 is a functional ligand for CX3CR1. J Immunol. 2010;185(11):6472–9. 10.4049/jimmunol.0904126 . 20974991

[36] Zhou L, Kawate T, Liu X, Kim YB, Zhao Y, Feng G, et al STAT6 phosphorylation inhibitors block eotaxin-3 secretion in bronchial epithelial cells. Bioorg Med Chem. 2012;20(2):750–8. 10.1016/j.bmc.2011.12.006 . 22217933

[37] Rokudai A, Terui Y, Kuniyoshi R, Mishima Y, Mishima Y, Aizu-Yokota E, et al Differential regulation of eotaxin-1/CCL11 and eotaxin-3/CCL26 production by the TNF-alpha and IL-4 stimulated human lung fibroblast. Biol Pharm Bull. 2006;29(6):1102–9. . 16755001 10.1248/bpb.29.1102

[38] Wang Y, Moreland M, Wagner JG, Ames BN, Illek B, Peden DB, et al Vitamin E forms inhibit IL-13/STAT6-induced eotaxin-3 secretion by up-regulation of PAR4, an endogenous inhibitor of atypical PKC in human lung epithelial cells. J Nutr Biochem. 2012;23(6):602–8. 10.1016/j.jnutbio.2011.03.003 21764283 PMC3201713

